# How to Rest

**Published:** 1893-09

**Authors:** 


### How to Rest.

To understand the way to rest, is of more importance than to know how to work. The latter can be learned easily; the former it takes years to learn, and some people never learn the art of resting. It is simply a change of scenes and activities. Loafing may not be resting. Sleeping is not always resting. Sitting down for days with nothing to do, is not restful. A change is needed to bring into play a different set of faculties, and to turn the life into a new channel. The man who works hard, finds his best rest in playing hard. The man who is burdened with care, finds relief in something that is active, yet free from responsibility. Above all, keep good-natured, and don't abuse your best friend, the stomach.

